# (*E*)-4-Bromo-*N*′-(2-chloro­benzyl­idene)benzohydrazide

**DOI:** 10.1107/S1600536809012860

**Published:** 2009-04-18

**Authors:** Xiao-Hong Shu, Yun-Peng Diao, Mo-Lin Li, Xu Yan, Jia Liu

**Affiliations:** a College of Pharmacy, Dalian Medical University, Liaoning 116044, People’s Republic of China; b College of Basic Medical Sciences, Dalian Medical University, Liaoning 116044, People’s Republic of China

## Abstract

In the title compound, C_14_H_10_BrClN_2_O, the dihedral angle between the two benzene rings is 11.4 (2)°. In the crystal structure, mol­ecules are connected *via* inter­molecular N—H⋯O hydrogen bonds into one-dimensional chains running parallel to the *c* axis.

## Related literature

For the biological activity of hydrazones and Schiff bases, see: Bhandari *et al.* (2008 ▶); Sinha *et al.* (2008 ▶). For a related structure, see: Pan & Yang (2005 ▶). For bond-length data, see: Allen *et al.* (1987 ▶).
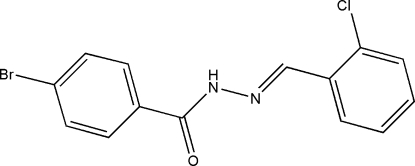


## Experimental

### 

#### Crystal data

C_14_H_10_BrClN_2_O
*M* *_r_* = 337.60
Monoclinic, 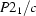

*a* = 11.218 (4) Å
*b* = 13.512 (5) Å
*c* = 9.200 (3) Å
β = 97.077 (6)°
*V* = 1383.9 (8) Å^3^
*Z* = 4
Mo *K*α radiation
μ = 3.16 mm^−1^
*T* = 298 K
0.23 × 0.20 × 0.20 mm

#### Data collection

Bruker SMART CCD diffractometer
Absorption correction: multi-scan (*SADABS*; Siemens, 1996 ▶) *T*_min_ = 0.491, *T*_max_ = 0.531
6907 measured reflections
2438 independent reflections
1948 reflections with *I* > 2σ(*I*)
*R*_int_ = 0.023

#### Refinement

*R*[*F* ^2^ > 2σ(*F* ^2^)] = 0.035
*wR*(*F* ^2^) = 0.094
*S* = 1.03
2438 reflections
172 parameters
H-atom parameters constrained
Δρ_max_ = 0.57 e Å^−3^
Δρ_min_ = −0.39 e Å^−3^

### 

Data collection: *SMART* (Siemens, 1996 ▶); cell refinement: *SAINT* (Siemens, 1996 ▶); data reduction: *SAINT*; program(s) used to solve structure: *SHELXS97* (Sheldrick, 2008 ▶); program(s) used to refine structure: *SHELXL97* (Sheldrick, 2008 ▶); molecular graphics: *SHELXTL* (Sheldrick, 2008 ▶); software used to prepare material for publication: *SHELXTL*.

## Supplementary Material

Crystal structure: contains datablocks global, I. DOI: 10.1107/S1600536809012860/hb2944sup1.cif

Structure factors: contains datablocks I. DOI: 10.1107/S1600536809012860/hb2944Isup2.hkl

Additional supplementary materials:  crystallographic information; 3D view; checkCIF report

## Figures and Tables

**Table 1:** Hydrogen-bond geometry (Å, °)

*D*—H⋯*A*	*D*—H	H⋯*A*	*D*⋯*A*	*D*—H⋯*A*
N2—H2⋯O1^i^	0.86	2.12	2.918 (3)	154

Symmetry code: (i) 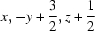.

**Fig. 1.**
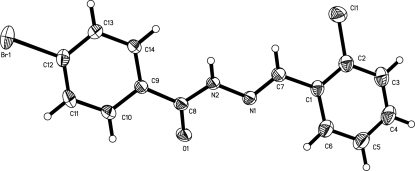
The molecular structure of (I), with displacement ellipsoids drawn at the 30% probability level.

**Fig. 2.**
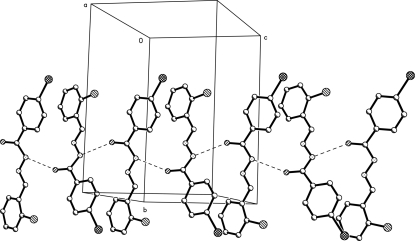
The one-dimensional chains structure along c axis. The donor···acceptor for the intermolecular hydrogen bonds are shown as dashed lines.

## supplementary crystallographic information

### Comment

Hydrazones and Schiff bases have attracted much attention for their excellent biological properties, especially for their potential pharmacological and antitumor properties (Bhandari *et al.*, 2008; Sinha *et al.*, 2008). In this paper, the crystal structure of the title compound, (I), a new Schiff base compound derived from the condensation reaction of 2-chlorobenzaldehyde with 4-bromobenzohydrazide is reported.

The Schiff base molecule of the compound displays a *trans* configuration with respect to the C=N and C—N bonds (Fig. 1). All the bond lengths are within normal ranges (Allen *et al.*, 1987), and are comparable to those in the related compound *N'*-(2-chlorobenzylidene)-2-hydroxybenzohydrazide (Pan & Yang, 2005). The dihedral angle between the two benzene rings is 11.4 (2)°. In the crystal structure, the C_14_H_10_BrClN_2_O molecules are connected *via* intermolecular N—H···O hydrogen bonds into one-dimensional chains running parallel to the *c* axis (Table 1 & Fig. 2).

### Experimental

2-Chlorobenzaldehyde (0.1 mmol) and 4-bromobenzohydrazide acid hydrazide (0.1 mmol) were dissolved in a 95% ethanol solution (10 ml). The mixture was stirred at room temperature to give a clear colorless solution. Colourless blocks of (I) were formed by gradual evaporation of the solvent over a period of five days at room temperature.

### Refinement

All H atoms were placed in geometrically idealized positions, with C—H = 0.93 Å and N—H = 0.86 Å. *U*_iso_(H) = 1.2*U*_eq_(C,*N*).

### Crystal data

**Table:**

C_14_H_10_BrClN_2_O	*F*(000) = 672
*M**_r_* = 337.60	*D*_x_ = 1.620 Mg m^−^^3^
Monoclinic, *P*2_1_/*c*	Mo *K*α radiation, λ = 0.71073 Å
Hall symbol: -P 2ybc	Cell parameters from 2695 reflections
*a* = 11.218 (4) Å	θ = 2.4–26.2°
*b* = 13.512 (5) Å	µ = 3.16 mm^−^^1^
*c* = 9.200 (3) Å	*T* = 298 K
β = 97.077 (6)°	Block, colorless
*V* = 1383.9 (8) Å^3^	0.23 × 0.20 × 0.20 mm
*Z* = 4	

### Data collection

**Table:**

Bruker SMART CCD diffractometer	2438 independent reflections
Radiation source: fine-focus sealed tube	1948 reflections with *I* > 2σ(*I*)
graphite	*R*_int_ = 0.023
ω scans	θ_max_ = 25.1°, θ_min_ = 1.8°
Absorption correction: multi-scan (*SADABS*; Siemens, 1996)	*h* = −13→13
*T*_min_ = 0.491, *T*_max_ = 0.531	*k* = −10→16
6907 measured reflections	*l* = −10→10

### Refinement

**Table:**

Refinement on *F*^2^	Primary atom site location: structure-invariant direct methods
Least-squares matrix: full	Secondary atom site location: difference Fourier map
*R*[*F*^2^ > 2σ(*F*^2^)] = 0.035	Hydrogen site location: inferred from neighbouring sites
*wR*(*F*^2^) = 0.094	H-atom parameters constrained
*S* = 1.03	*w* = 1/[σ^2^(*F*_o_^2^) + (0.0447*P*)^2^ + 0.9326*P*] where *P* = (*F*_o_^2^ + 2*F*_c_^2^)/3
2438 reflections	(Δ/σ)_max_ = 0.001
172 parameters	Δρ_max_ = 0.56 e Å^−^^3^
0 restraints	Δρ_min_ = −0.39 e Å^−^^3^

### Special details

**Table:**

Geometry. All e.s.d.'s (except the e.s.d. in the dihedral angle between two l.s. planes) are estimated using the full covariance matrix. The cell e.s.d.'s are taken into account individually in the estimation of e.s.d.'s in distances, angles and torsion angles; correlations between e.s.d.'s in cell parameters are only used when they are defined by crystal symmetry. An approximate (isotropic) treatment of cell e.s.d.'s is used for estimating e.s.d.'s involving l.s. planes.
Refinement. Refinement of *F*^2^ against ALL reflections. The weighted *R*-factor *wR* and goodness of fit *S* are based on *F*^2^, conventional *R*-factors *R* are based on *F*, with *F* set to zero for negative *F*^2^. The threshold expression of *F*^2^ > σ(*F*^2^) is used only for calculating *R*-factors(gt) *etc*. and is not relevant to the choice of reflections for refinement. *R*-factors based on *F*^2^ are statistically about twice as large as those based on *F*, and *R*- factors based on ALL data will be even larger.

### Fractional atomic coordinates and isotropic or equivalent isotropic displacement parameters (Å^2^)

**Table:**

	*x*	*y*	*z*	*U*_iso_*/*U*_eq_	
Br1	0.45713 (4)	1.17921 (3)	0.84354 (5)	0.07653 (18)	
Cl1	0.23484 (10)	0.37589 (7)	0.63350 (10)	0.0790 (3)	
N1	0.1872 (2)	0.66418 (16)	0.4378 (2)	0.0423 (5)	
N2	0.2302 (2)	0.74288 (16)	0.5254 (2)	0.0419 (5)	
H2	0.2331	0.7398	0.6191	0.050*	
O1	0.2623 (2)	0.83236 (15)	0.3250 (2)	0.0555 (6)	
C1	0.1301 (3)	0.4950 (2)	0.4158 (3)	0.0439 (7)	
C2	0.1495 (3)	0.3987 (2)	0.4655 (3)	0.0521 (7)	
C3	0.1046 (3)	0.3167 (2)	0.3829 (4)	0.0634 (9)	
H3	0.1184	0.2529	0.4190	0.076*	
C4	0.0401 (3)	0.3319 (3)	0.2483 (4)	0.0674 (10)	
H4	0.0100	0.2781	0.1924	0.081*	
C5	0.0197 (3)	0.4264 (3)	0.1954 (4)	0.0670 (10)	
H5	−0.0245	0.4358	0.1041	0.080*	
C6	0.0637 (3)	0.5072 (3)	0.2759 (4)	0.0558 (8)	
H6	0.0497	0.5705	0.2378	0.067*	
C7	0.1768 (3)	0.5816 (2)	0.5014 (3)	0.0448 (7)	
H7	0.1987	0.5760	0.6019	0.054*	
C8	0.2680 (3)	0.82507 (19)	0.4591 (3)	0.0402 (6)	
C9	0.3160 (2)	0.90782 (19)	0.5568 (3)	0.0387 (6)	
C10	0.3159 (3)	1.0028 (2)	0.4976 (4)	0.0639 (10)	
H10	0.2873	1.0121	0.3993	0.077*	
C11	0.3572 (4)	1.0832 (2)	0.5811 (4)	0.0687 (10)	
H11	0.3549	1.1463	0.5404	0.082*	
C12	0.4020 (3)	1.0687 (2)	0.7257 (3)	0.0485 (7)	
C13	0.4055 (3)	0.9757 (2)	0.7869 (3)	0.0502 (7)	
H13	0.4371	0.9667	0.8844	0.060*	
C14	0.3620 (3)	0.8957 (2)	0.7029 (3)	0.0432 (6)	
H14	0.3635	0.8330	0.7447	0.052*	

### Atomic displacement parameters (Å^2^)

**Table:**

	*U*^11^	*U*^22^	*U*^33^	*U*^12^	*U*^13^	*U*^23^
Br1	0.0834 (3)	0.0523 (2)	0.0923 (3)	−0.02495 (18)	0.0047 (2)	−0.02263 (19)
Cl1	0.1214 (8)	0.0504 (5)	0.0598 (6)	0.0029 (5)	−0.0099 (5)	0.0011 (4)
N1	0.0549 (14)	0.0366 (13)	0.0344 (12)	0.0020 (10)	0.0014 (10)	−0.0050 (10)
N2	0.0650 (15)	0.0336 (12)	0.0262 (11)	−0.0013 (10)	0.0018 (10)	−0.0021 (9)
O1	0.0959 (17)	0.0437 (12)	0.0263 (11)	0.0018 (11)	0.0054 (10)	0.0010 (8)
C1	0.0496 (16)	0.0391 (15)	0.0445 (16)	−0.0057 (12)	0.0120 (13)	−0.0079 (13)
C2	0.0590 (19)	0.0442 (17)	0.0542 (18)	−0.0014 (14)	0.0108 (15)	−0.0081 (14)
C3	0.076 (2)	0.0392 (17)	0.077 (3)	−0.0050 (15)	0.017 (2)	−0.0117 (16)
C4	0.076 (2)	0.058 (2)	0.066 (2)	−0.0167 (18)	0.0048 (19)	−0.0232 (18)
C5	0.069 (2)	0.071 (2)	0.058 (2)	−0.0143 (18)	−0.0046 (17)	−0.0136 (18)
C6	0.0590 (19)	0.0543 (19)	0.0540 (19)	−0.0083 (15)	0.0061 (15)	−0.0098 (15)
C7	0.0588 (18)	0.0397 (16)	0.0360 (15)	−0.0021 (13)	0.0058 (13)	−0.0027 (12)
C8	0.0519 (17)	0.0352 (14)	0.0332 (15)	0.0075 (12)	0.0038 (12)	0.0004 (11)
C9	0.0510 (16)	0.0333 (14)	0.0322 (14)	0.0015 (12)	0.0067 (12)	−0.0004 (11)
C10	0.105 (3)	0.0416 (17)	0.0414 (18)	−0.0093 (17)	−0.0061 (17)	0.0104 (14)
C11	0.103 (3)	0.0325 (17)	0.068 (2)	−0.0132 (17)	0.001 (2)	0.0098 (16)
C12	0.0519 (17)	0.0397 (16)	0.0542 (19)	−0.0110 (13)	0.0078 (14)	−0.0071 (14)
C13	0.0638 (19)	0.0464 (17)	0.0384 (16)	−0.0065 (14)	−0.0024 (14)	−0.0024 (13)
C14	0.0570 (17)	0.0341 (14)	0.0381 (15)	−0.0007 (12)	0.0042 (12)	0.0041 (12)

### Geometric parameters (Å, °)

**Table:**

Br1—C12	1.903 (3)	C5—C6	1.377 (4)
Cl1—C2	1.742 (3)	C5—H5	0.9300
N1—C7	1.272 (4)	C6—H6	0.9300
N1—N2	1.385 (3)	C7—H7	0.9300
N2—C8	1.359 (3)	C8—C9	1.493 (4)
N2—H2	0.8600	C9—C14	1.388 (4)
O1—C8	1.232 (3)	C9—C10	1.394 (4)
C1—C2	1.388 (4)	C10—C11	1.378 (5)
C1—C6	1.415 (4)	C10—H10	0.9300
C1—C7	1.470 (4)	C11—C12	1.377 (5)
C2—C3	1.401 (4)	C11—H11	0.9300
C3—C4	1.371 (5)	C12—C13	1.376 (4)
C3—H3	0.9300	C13—C14	1.383 (4)
C4—C5	1.375 (5)	C13—H13	0.9300
C4—H4	0.9300	C14—H14	0.9300
			
C7—N1—N2	116.8 (2)	N1—C7—H7	120.1
C8—N2—N1	118.2 (2)	C1—C7—H7	120.1
C8—N2—H2	120.9	O1—C8—N2	122.3 (3)
N1—N2—H2	120.9	O1—C8—C9	120.9 (2)
C2—C1—C6	116.9 (3)	N2—C8—C9	116.8 (2)
C2—C1—C7	122.6 (3)	C14—C9—C10	117.9 (3)
C6—C1—C7	120.5 (3)	C14—C9—C8	123.9 (2)
C1—C2—C3	122.1 (3)	C10—C9—C8	118.1 (2)
C1—C2—Cl1	120.3 (2)	C11—C10—C9	121.6 (3)
C3—C2—Cl1	117.5 (3)	C11—C10—H10	119.2
C4—C3—C2	119.1 (3)	C9—C10—H10	119.2
C4—C3—H3	120.5	C12—C11—C10	118.9 (3)
C2—C3—H3	120.5	C12—C11—H11	120.5
C3—C4—C5	120.3 (3)	C10—C11—H11	120.5
C3—C4—H4	119.8	C13—C12—C11	121.0 (3)
C5—C4—H4	119.8	C13—C12—Br1	119.5 (2)
C4—C5—C6	120.9 (3)	C11—C12—Br1	119.5 (2)
C4—C5—H5	119.5	C12—C13—C14	119.6 (3)
C6—C5—H5	119.5	C12—C13—H13	120.2
C5—C6—C1	120.7 (3)	C14—C13—H13	120.2
C5—C6—H6	119.7	C13—C14—C9	120.9 (3)
C1—C6—H6	119.7	C13—C14—H14	119.6
N1—C7—C1	119.9 (3)	C9—C14—H14	119.6
			
C7—N1—N2—C8	165.4 (3)	N1—N2—C8—C9	−178.9 (2)
C6—C1—C2—C3	−0.9 (5)	O1—C8—C9—C14	−157.9 (3)
C7—C1—C2—C3	180.0 (3)	N2—C8—C9—C14	22.8 (4)
C6—C1—C2—Cl1	177.7 (2)	O1—C8—C9—C10	21.4 (4)
C7—C1—C2—Cl1	−1.4 (4)	N2—C8—C9—C10	−158.0 (3)
C1—C2—C3—C4	0.5 (5)	C14—C9—C10—C11	−1.6 (5)
Cl1—C2—C3—C4	−178.1 (3)	C8—C9—C10—C11	179.1 (3)
C2—C3—C4—C5	−0.2 (6)	C9—C10—C11—C12	1.4 (6)
C3—C4—C5—C6	0.3 (6)	C10—C11—C12—C13	−0.1 (6)
C4—C5—C6—C1	−0.7 (6)	C10—C11—C12—Br1	−179.0 (3)
C2—C1—C6—C5	1.0 (5)	C11—C12—C13—C14	−1.0 (5)
C7—C1—C6—C5	−179.9 (3)	Br1—C12—C13—C14	177.9 (2)
N2—N1—C7—C1	179.2 (2)	C12—C13—C14—C9	0.8 (5)
C2—C1—C7—N1	160.0 (3)	C10—C9—C14—C13	0.5 (5)
C6—C1—C7—N1	−19.1 (4)	C8—C9—C14—C13	179.7 (3)
N1—N2—C8—O1	1.8 (4)		

### Hydrogen-bond geometry (Å, °)

**Table:**

*D*—H···*A*	*D*—H	H···*A*	*D*···*A*	*D*—H···*A*
N2—H2···O1^i^	0.86	2.12	2.918 (3)	154

Symmetry codes: (i) *x*, −*y*+3/2, *z*+1/2.
